# Outdoor Activity in the Daytime, but Not the Nighttime, Predicts Better Mental Health Status During the COVID-19 Curfew in the United Arab Emirates

**DOI:** 10.3389/fpubh.2022.829362

**Published:** 2022-04-04

**Authors:** Fatme Al Anouti, Justin Thomas, Spyridon Karras, Nour El Asswad

**Affiliations:** ^1^Department of Health Sciences, College of Natural and Health Sciences, Zayed University, Abu Dhabi, United Arab Emirates; ^2^Department of Psychology, College of Natural and Health Sciences, Zayed University, Abu Dhabi, United Arab Emirates; ^3^Division of Endocrinology and Metabolism, First Department of Internal Medicine, Medical School, Aristotle University of Thessaloniki, AHEPA University Hospital, Thessaloniki, Greece; ^4^School of Global Health, McMaster University, Hamilton, ON, Canada

**Keywords:** COVID-19, depressive symptoms, somatization, vitamin D, United Arab Emirates

## Abstract

**Background:**

The COVID-19 pandemic and the associated infection prevention and control measures had a negative impact on the mental health of many people. In the United Arab Emirates (UAE), infection control measures implemented after March 24th, 2020, placed necessary restrictions on people's freedom of movement.

**Aim:**

This study aimed to assess the association between levels of daytime vs. nighttime outdoor activity and mental health among a sample of UAE residents during the lockdown period.

**Method:**

An opportunity sample of 245 participants completed an online survey assessing levels of depression, somatic symptoms, daytime and nighttime activity levels.

**Results:**

Multivariate logistic regression revealed that daytime activity, but not nighttime activity, was associated with a lower risk of clinically significant depressive and somatic symptomatology.

**Conclusion:**

The association of better mental health with daytime not nighttime outdoor activity could be possibly attributed to vitamin D, but further studies are needed to confirm this speculation.

## Introduction

The COVID-19 pandemic disrupted many aspects of routine life, adversely impacting physical and mental health, social activity, and economic status (1). Once the World Health Organization declared the disease a global pandemic in March 2020, many countries began implementing stringent infection prevention and control measures. Attempting to curtail the transmission of the virus and to prevent deaths and the catastrophic over-burdening of their healthcare systems, several governments including that of the United Arab Emirates (UAE) UAE, placed restriction on freedom of movement via the implementation of population-wide curfews and lockdowns (2, 3). The UAE enforced mandatory masking, social distancing and quarantine, suspended air travel, and closed all educational and public entities, except for vital services such as supermarkets, pharmacies, and healthcare facilities. Additionally, the UAE implemented a nation-wide 8 pm to 6 am curfew, requiring people to remain in their homes or face significant financial penalties.

The pandemic and the above-mentioned infection prevention and control measures have adversely affected mental health (4). At the most basic level, the grief related to COVID-19 deaths, and the fear of infection are highly likely to lead to an elevated risk of developing an associated mental health problem. In addition to the direct fear of the virus, the essential limitations on the freedom-of-movement, including social/physical distancing and quarantine, can also have undesirable impacts on psychological wellbeing (4).

Recent research across different countries worldwide and in the UAE have reportednegative changes in diet during the lockdown including consuming more calories, eating more snacks and less of fresh fruits and vegetables, and hence gaining weight (5–7). In addition, physical activity levels; which were already low in the UAE before COVID-19; have been reduced and negatively impacted by quarantine (8). COVID-19 had also increased the level of anxiety and depressive mental health symptoms drastically and unfavorably affected people's sleeping patterns along with dietary habits and physical activity levels (4, 9).

Several researchers have recently highlighted that the immune system could be supported by important micronutrients which could lower the risk of COVID-19 infection (10). Among those, vitamin D is the most attractive for research. An adequate intake of vitamins is crucial to fight against infections and inflammations, especially during COVID-19 pandemic. One important vitamin is vitamin D, which is well known to boost the immune system and to modulate the body's response to infections (11). Therefore, in such critical times a healthy balanced lifestyle containing vitamins and minerals is essential to overcome Covid19 and strengthen immunity. When infected by COVID-19 virus, the body increases the release of pro-inflammatory cytokines and C-reactive protein (12, 13). Vitamin D reduces the severity of the COVID-19 infection by minimizing the cytokine storm caused by the innate system. Based on the most recent evidence, vitamin D supplementation can reduce risk and severity of respiratory infections (14). Casual sun exposure by individuals could secure adequate vitamin D levels (15, 16). There is sufficient evidence during the COVID-19 pandemic to support recommending vitamin D given that many people are spending more time indoors and may not get the sufficient amount of vitamin D they need for optimal bone and overall health (17).

In this study, we aimed to examine the relationship between outdoor physical activity levels (daytime vs. nighttime) and mental health during COVID-19 lockdown among an opportunity sample of citizens and residents of the UAE. Our research could contribute important findings about mental health in the UAE and guide future intervention studies given the high prevalence of depression in the gulf region (18).

## Methods

### Study Design

This cross-sectional study utilized an online survey to assess mental health (depressive and somatic symptomatology) in a community sample of adults (*N* = 245) in the UAE between April 8th and April 18th, 2020. Respondents completed standardized assessments for symptom measures of depression and somatization, along with psychosocial and demographic variables including daytime and nighttime physical activity levels that might potentially influence such symptoms. Moreover, data about the use of vitamin D supplements during the Curfew was recorded. Bivariate and multivariate associations were calculated for the main study variables.

### Recruitment of Participants and Procedure

A-priori sample size estimates for regression analysis (19) with a medium effect size suggested a sample of size of 103. The final sample of participants (*N* = 245) were an opportunity sample recruited through the social media and email networks of UAE's National Program for Happiness and Wellbeing. For inclusion, participants were required to be adult (at least 18 years old) residents/citizens of the UAE residing in the country during April 2020. Participants who did not complete the battery fully (*n* = 8) were excluded. The final sample included both genders (199 females and 46 males) and broadly reflected the multiethnic and international nature of the UAE population. Ages ranged from 18 to 73 with a mean age of 33.47 ± 12.88. All participants undertook a survey that involved questions related to mental health status assessment (depression and somatization), demographics and psychosocial variables.

The survey was a self-report survey [rarely vs. frequently (yes/no), do you do this?]. All demographic and COVID-related measures were available in the two main languages commonly used in the UAE, namely Arabic and English. The developed survey was part of a larger ongoing national survey exploring the impact of COVID-19 in the UAE. Ethical clearance was obtained from Zayed University Research Ethics Committee (R201213) and from the Ministry of Health and Prevention's Research Ethics Committee (MOHAP/DXB-REC/ MMM/No. 49/2020). Data collection took place online and all participants consented prior to answering the survey.

### Psychosocial and Demographic Variables

The COVID-related items were adapted from Shevlin et al. (20). Moreover, some items related to outdoor activity were adapted from the previously validated Sun Avoidance Inventory which was an indicator of vitamin D lifestyle related factors among the UAE population (21–23). The questions were related to being on outdoor balcony, in a garden or yard, on a roof or terrace, walking or exercising outdoors. All items were duplicated for both daytime and nighttime sections.

### The Patient Health Questionnaire-8 (PHQ-8)

The PHQ-8 (24) is a commonly utilized standardized tool for assessing the prevalence and severity of depressive symptoms. The psychometric properties of the PHQ-8 scores had been widely supported and it consists of eight questions that examine the frequency of depressive symptoms over past 2 weeks. Responses can range from 0 to 3 (0 = not at all, 1 = several days, 2 = more than half the days, 3 = nearly every day). Total added scores range from 0 to 24 with scores below 5 indicating absence of considerable depressive symptoms. The presence of moderate depression (clinically significant depressive symptoms) was noted when the cut-off score of ≥10 was exceeded up to 14 as scores of 15 and above indicated severe levels of depression. The reliability of the scale among the current sample was excellent (α = 0.915).

### The Patient Health Questionnaire-15 (PHQ-15)

We used the PHQ15 to assess somatic symptoms (25). The inclusion of this measure follows the finding that psychological symptoms are often somatized, some individuals are more likely to experience or report distress in somatic terms (vague aches and pains, insomnia, headaches etc.,) rather than the psychological ones (26). Previous research suggests that among Arab populations depression is very frequently described in terms of psychosomatic symptoms (27). The PHQ-15 is a 15-item self-report measure, asking respondents about the degree of distress in relation to physical health problems (insomnia, headaches, stomach aches etc.) over the last 2 weeks. All items are rated on a 3-point Likert scale: 0 (not bothered at all) to 2 (bothered a lot). We excluded the item focused on ‘menstrual problems' due to its gender-specific content. The total scale scores were used, where scores of 10 and above are viewed as clinically significant. Multiple previous studies attest to the reliability and validity of the PHQ-15 (28).

### Timeline of the Study

The United Arab Emirates (UAE) enacted several infection prevention and control measures, including social distancing, quarantine, curfews, the closure of educational/recreational facilities, and the cessation of in and outbound passenger air travel. Supplementary Table 1, based on UAE governmental sources, provides a timeline of the nation's primary infection prevention and control measures around the time that this study was planned and undertaken. Adapted from Thomas et al., 2020 (29).

### Statistical Analysis

Bivariate and multiple logistic regressions were done with R (R Core Team, 2020), using generalized linear models in the base package. Two binary logistic regression models were used to predict Somatization (PHQ-15) and Depression (PHQ-8), computing bivariate odds ratios (OR) and multivariate adjusted odds ratios (AOR) for all predictor variables. The predictor variables were age, gender, citizenship, outdoor activity (day and night) and VTD supplement use.

## Results

### Outdoor Activity

Daytime outdoor activity levels (*M* = 3.97 SD = 3.72) were higher than nighttime activity levels (*M* = 3.51, SD = 3.96); indicating that participants were more engaged in daytime outdoor activity as compared to nighttime. This difference was statistically significant *t* (241) = 1.86, *p* = 0.032. Table 1 details the mean score for each outdoor activity. Walking outdoors was the most highly endorsed outdoor activity for both daytime and nighttime.

**Table 1:** Means and standard deviations for endorsement of outdoor activities.

**Outdoor activities**	**Daytime**		**Nighttime**	
	***M***	**SD**	***M***	**SD**
Being on an outdoor balcony	0.93	1.15	0.84	1.12
Being in a garden or yard	0.99	1.17	0.78	1.09
Walking outdoors	1.00	1.15	0.89	1.09
Being on a roof or outdoor terrace	0.49	0.92	0.54	0.99
Outdoor exercise	0.56	0.98	0.49	0.94

* *SD, Standard Deviation*.

### Vitamin D Supplementation

In response to the question “To reduce your risk of being infected by the coronavirus COVID-19 have you recently taken vitamin D supplement”. Participants gave the following responses No (*N* = 142, 56%), Occasionally (*N* = 38, 15.2%) and Regularly (*N* = 67, 26.8%).

### Psychopathology Scores

Psychopathology scores were elevated with 47.95% of the participants scoring above the cut-off of the PHQ-8 for depressive symptoms. Similarly, high levels of clinically significant somatic symptomatology were also observed with 34.31% of participants scoring above the cut-off of the PH-Q15 for somatic symptoms. Table 2 details the mean score for psychopathology symptoms of depression and somatization for the study participants.

**Table 2:** Means and standard deviations for psychopathology symptom measures.

	**M**	**SD**	**% Above the cut-off**
Depression	9.93	7.30	47.95
Somatization	7.56	5.77	34.31

* *SD, Standard Deviation*.

### Correlational Analysis

Only outdoor activity in the daytime was associated with lower levels of depression and somatization as revealed by the significant negative correlation with PH-Q8 depression and PH-Q15 somatization scores (*p* <.01 and *p* <.001, respectively). Table 3 details the bivariate correlations between the study's key variables. Nighttime outdoor activity and use of vitamin D supplements however were uncorrelated with levels of psychopathology.

**Table 3:** Correlations for all the main variables of the study.

	**Depression**	**VTD suppl**.	**Day activity**	**Nigh activity**
Somatization	0.659^***^	−0.043	−0.195^***^	−0.088
Depression		−0.078	−0.189^**^	−0.109
VTD suppl.			0.016	0.100
Day activity				0.646^***^

* *p < 0.05*,

** *p < 0.01*,

*** *p < 0.001*.

The results of bivariate and multiple logistic regression analyses to predict likelihood of depression (PHQ-8) in Table 4 reveal the significance of prediction with daytime outdoor activity levels (*p* < 0.01).

**Table 4:** Bivariate and multivariate logistic regression predicting depressive symptoms.

		**Score above** **PHQ8 cut-off**	**Odds ratio**	**Adjusted odds ratio**
	***N***	***N*** **(%)**		
**Age below 30**				
No	125	39 (31.2%)	-	-
Yes	120	81 (67.5%)	4.587 (2.673–7.812)^***^	4.032 (2.309–7.042)^***^
**Gender**				
Male	46	12 (26.0%)	-	-
Female	199	108 (54.2%)	3.363 (1.645–6.872)^***^	2.531 (1.167–5.491)^**^
**UAE citizen**				
No	91	32 (35.1%)	-	-
Yes	154	88 (57.1%)	2.458 (1.439–4.201)^***^	0.937 (0.463–1.896)
**Day active**				
Yes	70	23 (22.8%)	-	-
No	175	97 (55.4%)	2.541 (1.421–4.543)^**^	2.458 (1.308–4.619)^**^
**Night active**				
Yes	64	27 (42.1%)	-	-
No	181	93 (51.3%)	1.448 (0.815–2.575)	
**VTD supplement**				
No	141	74 (44.2%)	-	-
Yes	104	46 (52.4%)	1.393 (0.837–2.317)	

* *p < 0.05*,

** *p < 0.01*,

*** *p < 0.001*.

**Figure 1:**
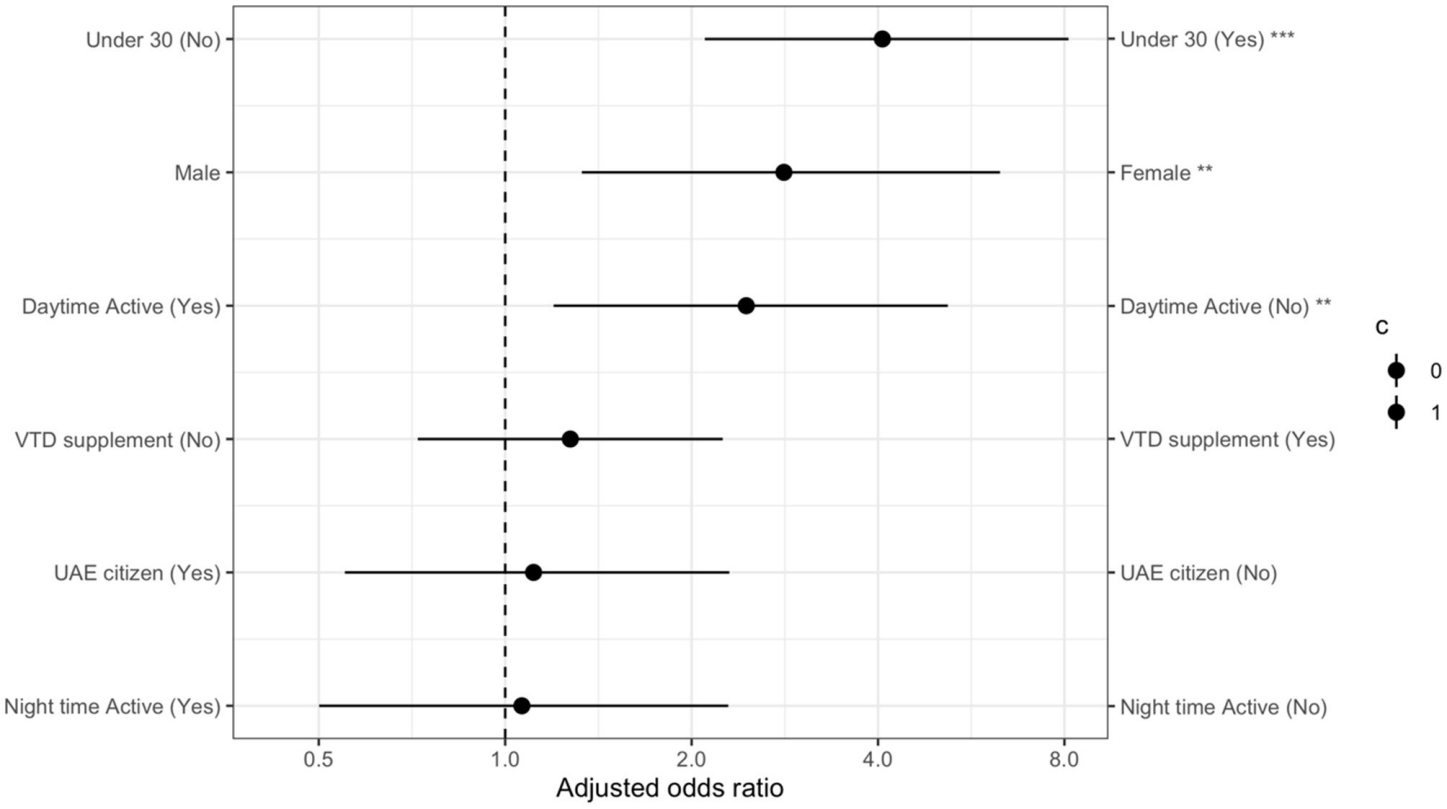
Multivariate logistic regression model analysis for higher risk of clinically significant depressive symptoms with adjusted odds ratios for all variables.

Low levels of daytime outdoor activity were associated with higher risk of clinically significant depressive symptoms. This effect remained even after controlling for age, gender and citizenship status. Figure 1 details the AOR for all variables in the multivariate logistic regression model.

The results of the bivariate and multivariate analysis for the risk of developing clinically significant levels of somatic symptoms are shown in Table 5. The data demonstrates the significance of prediction with only daytime outdoor activity levels (*p* < 0.05).

**Table 5:** Bivariate and multivariate logistic regression predicting somatic symptoms.

		**Score above** **PHQ15 cut-off**	**Odds ratio**	**Adjusted odds ratio**
	***N***	***N*** **(%)**		
**Under 30**				
No	119	22 (18.4%)	-	-
Yes	120	59 (49.1%)	4.273 (2.375–7.633)^***^	3.690 (1.782–7.633)^***^
**Gender**				
Male	46	4 (8.6%)	-	-
Female	193	77 (39.8%)	6.970 (2.403– 20.218)^***^	5.505 (1.1827–16.58)^**^
**Citizen**				
No	87	19 (21.8%)	-	-
Yes	152	62 (42.7%	2.465 (1.349–4.505)^**^	0..941 (0.429–2.061)
**Day active**				
Yes	67	15 (22.3%)	-	-
No	172	66 38.3%)	2.158 (1.125–4.141)^*^	2.119 (1.053–4.265)^*^
**Night active**				
Yes	62	19 (30.6%)	-	-
No	177	62 (35.0%)	1.220 (0.655–2.273)	
**VTD supplement**				
Yes	101	32 (31.6%)	-	-
No	138	49 (35.5%)	1.187 (0.688–2.048)	

* *p < 0.05*,

** *p < 0.01*,

*** *p < 0.001*.

**Figure 2:**
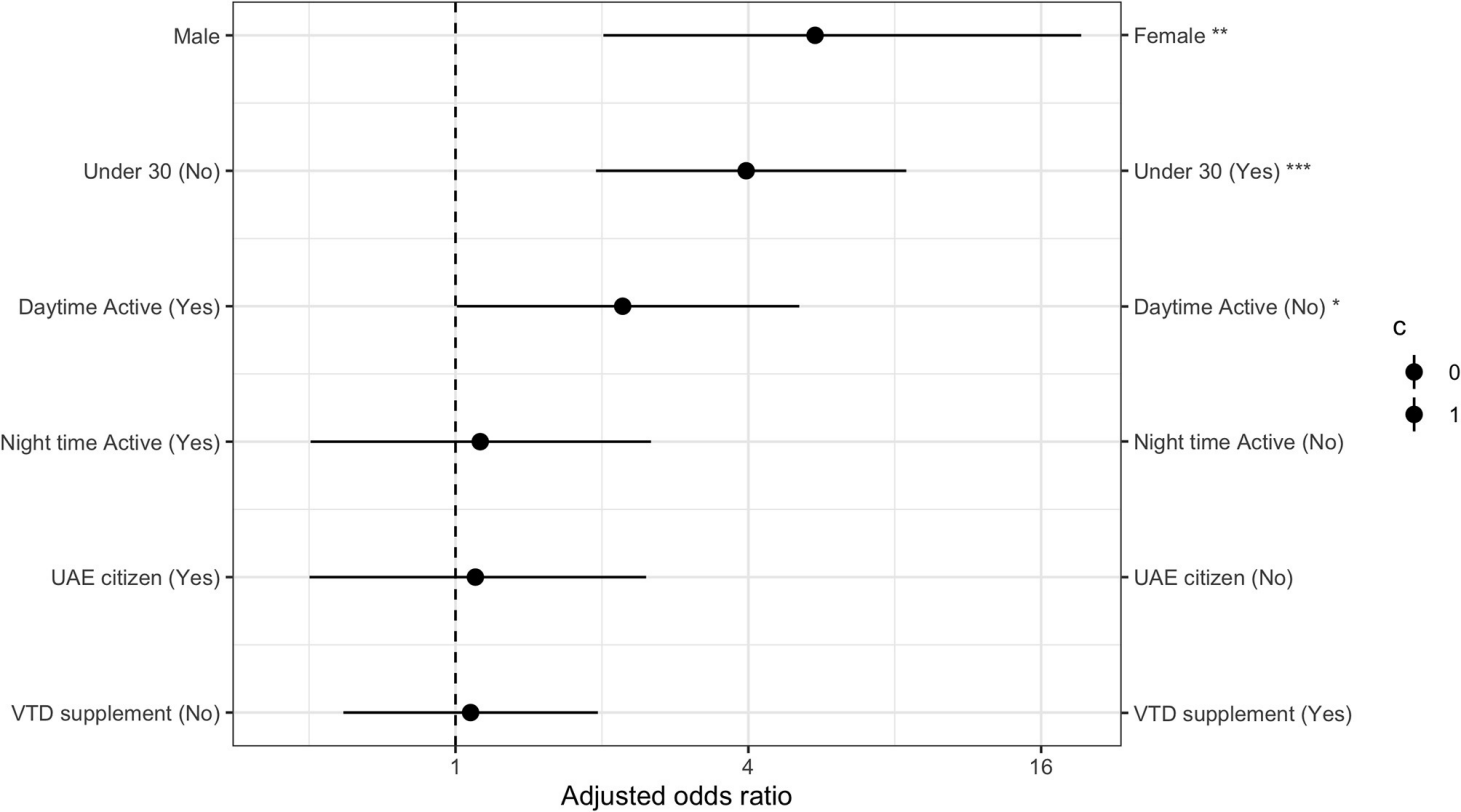
Multivariate logistic regression model analysis for higher risk of clinically significant somatic symptoms with adjusted odds ratios for all variables.

Low levels of daytime outdoor activity were also associated with higher risk of clinically significant somatic symptoms. This effect remained even after controlling for age, gender, and citizenship status. Figure 2 details the AOR ratios for all variables in the multivariate logistic regression model.

## Discussion

The study results suggest that mental health among the UAE population had been adversely affected during the COVID-19 curfew period. Psychopathology scores were elevated beyond pre-pandemic norms, with close to half the participants scoring above the cut-off of the PHQ-8. Similarly, high levels of somatic symptomatology were also reported on the PHQ-15. Such results during a time of crisis were expected since the current COVID-19 pandemic has influenced the psychological health of individuals all over the world due to the necessary infection-prevention and control measures, such as lockdowns. Several other studies concur, each reporting alarmingly elevated rates of mental conditions during the COVID-19 (30–32) and worsened clinical symptoms for those who already suffered from mental conditions (33).

In the present study, citizens (Emirati nationals) generally reported higher rates of depression and somatization compared to non-Emirati residents. They were also more likely to score above the PHQ8 and PHQ15 cut-offs. This effect, however, disappears when we adjust for other variables (age in particular). The Emirati section of the sample is significantly younger than the non-Emirati resident counterpart, hence age rather than nationality is associated with elevated symptoms of somatization and depression. Similar age-related findings had been reported in other national (34) and international studies (20) undertaken around the same time.

It had been widely demonstrated that moderate to vigorous physical activity can improve overall health, encompassing both physical and mental wellbeing (35). Ample research has shown that physical activity could enhance sleep quality and reduce risks of developing stress, anxiety and depression as it increases the level of endorphins, dopamine, adrenaline, myokines, serotonin and endocannaboids (36, 37). meta A meta-analysis of prospective studies encompassing 1,837,794 person-years concluded that physical activity was a protective factor against depression across different age groups and geographical locations across the world (38). Similarly, physical activity was shown to confer protection against anxiety after examining evidence from studies with 3,57,424 person-years (39).

During COVID-19 pandemic, physical activity had been reported to be associated with less depression and anxiety across different parts of the globe (40). Conversely, the reduction in physical activity levels due to COVID-19 lockdowns had a negative impact on overall health and wellbeing (41–45). The findings in our study are in line with reports from other studies examining the life style behaviors of individuals in the Middle East and North Africa (MENA) region during quarantine (46). A study by Cheikh Ismail et al. (2020) demonstrated that the UAE population had experienced higher levels of anxiety and depressive mental health symptoms during COVID-19 along with unfavorable sleeping patterns, eating habits, and physical activity levels (47). A similar study by Abouzid et al., (2021), showed a remarkable increase in sedentary lifestyle and reduction in physical activities due to people's tendency to spend more time on social media and television during quarantine (46).

In our current study, daytime activity, but not nighttime activity, was significantly correlated with lower depressive and somatic symptoms. Multivariate regression analysis revealed that outdoor physical activity; during the day; was a strong predictor of both depression and somatization and that such power remained upon adjustment for other variables. During the COVID-19 pandemic, confinement and spending more time indoors restricted outdoor physical activity and shifted the life styles of many individuals toward being more sedentary. Such changes were associated with a reduced ability to fight the viral infection and also negative effects on the brain health and immune system (48). Specific Outdoor physical activity in natural environments was correlated with reduced odds of depression when compared to inactivity and indoor activity among 88,522 individuals (49). Although the mechanism behind this finding is not fully elucidated, it is suggested that exposure to sunlight and bonding with nature might be a contributing factor since it could create a more pleasant experience (50). A systematic review conducted by Thompson et al., (2011) tested the hypothesis that there are additional benefits attained from performing physical activity outdoors in natural spaces vs. indoor settings. The review demonstrated some additional positive effects on self-reported mental wellbeing immediately following exercise in nature which are not seen following the same exercise indoors (51).

Our findings regarding the robust correlation of lower levels of depression and somatization with daytime but not nighttime outdoor physical activity could be interpreted in the context of other results from previous research in the UAE regarding depression, vitamin D deficiency and sun avoidance which demonstrated that high levels of sun avoidance are associated with higher risk of both depression and vitamin D deficiency (22, 23). The fact that nighttime outdoor activity levels did not show a correlation with depressive and somatic symptoms further makes this suggestion about a possible role for vitamin D more plausible.

Good nutrition and active lifestyle are the two chief elements in maintaining a good immunity to fight the SARS-CoV-2 virus. However, outdoor activity and access to healthy food have been impaired across several countries by either total or partial lockdowns during the COVID-19 pandemic. This had consequently reduced people's intake of vitamins and minerals and their sunlight exposure. Ultraviolet B (UV-B) light from sunlight exposure is the main factor for producing the active form of vitamin D in the body (52). As more than 80% of the body's requirement of vitamin D is supplied from UV-B sunlight exposure (53, 54) the lowered levels of outdoor activities due to COVID-19 lockdowns might hence contribute to a higher prevalence of vitamin D deficiency (55).

Vitamin D has an important physiological role in the brain including brain development, synaptic plasticity, neuroprotection, neurotransmission, and neuroimmunomodulation (56). studies have found low levels of vitamin D in patients with major depressive disorder (MDD) and bipolar disorder (BD) (57–59); however, its clinical and therapeutic effects on mental health is still under study (60, 61).

Interestingly, there was an increase in the intake of dietary supplements during the COVID-19 pandemic, particularly vitamin D supplement intake (increase rate of 31.8%) (46). In our study, we did not determine the increase rate prior to COVID-19, however 31.6% of the participants were taking Vitamin D (D3) supplements regularly/occasionally during the pandemic. The available over the counter dose in the country ranges from 1,000 to 50,000 IU for vitamin D. Previous studies from the UAE reported a low level of dietary and supplementary intake for vitamin D among the UAE population prior to COVID-19 (22, 62–64). Despite the abundance of sunshine in the UAE, there is an alarmingly high prevalence (around 80% among the population) for vitamin-D deficiency due to multiple reasons including cultural sartorial, religious and habitual which mainly reduce sun exposure and limit obtaining adequate vitamin D through UV-B (63).

A study by Di Nicola et al., 2020, showed that low 25-hydroxyvitamin D serum levels were significantly correlated with greater mental disturbance in patients with mood illnesses during the COVID-19 pandemic (65). Similar results were reported by Chen et al. demonstrated a significant association between low serum 25 (OH) D levels and poor mental health in a large sample of young adults (66). Moreover, many studies have found some positive impacts of vitamin D supplementation in individuals with clinical depression and vitamin D deficiency (60, 61, 67). Despite the positive associations obtained from observational studies, a large longitudinal randomized trial that has recently studied the effect of treatment with vitamin D3 supplementation on the rate and frequency of depression didn't achieve any significant difference in clinically relevant depressive symptoms or mood scores (61). This warrants further investigation and additional trials using different dosages and regimen.

### Strengths and Limitations

In this study, we propose a preliminary understanding of the psychosocial factors associated with elevated levels of depression and somatization among the UAE population during COVID-19 pandemic in the context of daytime vs. outdoor activity levels. To our knowledge, this is the first study in the UAE to ever examine the relationship between outdoor physical activity levels (daytime vs. nighttime) and mental health during COVID-19 lockdown among citizens and residents of the UAE. Such studies aiming to assess the impact of the pandemic on mental health and wellbeing are essential in order to fully decipher the implications and outcomes and pave way for the development of future intervention programs.

Despite the interesting results of this study, there are several important limitations. Given the need for a prompt data collection method to complete the study within a critical period during a rapidly evolving pandemic, we resorted to snowball and convenience sampling using internet and social media platform to invite participants. Such a sampling method might introduce bias and limit the generalizability of the findings. Hence, the results might not be fully projected to be representative of all the UAE population. Moreover, the serum 25 (OH) D levels which are frequently used as clinical indicators for the assessment of vitamin D status had not been measured. Another limitation pertains to data about sun exposure which was estimated by outdoor activity, however, sartorial style during exposure had not been recorded. Nevertheless, the findings of our study should encourage other researchers to further investigate the role of vitamin D as a potential resilience factor associated with psychological wellbeing during COVID-19 pandemic and recovery phase.

## Conclusion

Our study attempted to explore the impact of COVID-19 on mental health of population in the UAE. The data in our study had demonstrated that daytime outdoor activity levels during COVID-19 curfew in the UAE were significantly associated with lower depressive and somatic symptoms. Nighttime outdoor activity levels in contrast where not correlated with depression and somatic symptoms. We speculate that differences in results between daytime and nighttime outdoor activities could be attributed tothe positive effects of bonding with nature and exposure to sun which raises the levels of vitamin D. Such assumption however, requires much further investigation and additional research.

## Data Availability Statement

The raw data supporting the conclusions of this article will be made available by the authors, without undue reservation.

## Ethics Statement

The studies involving human participants were reviewed and approved by Zayed University and Ministry of Health. The patients/participants provided their written informed consent to participate in this study. Ethical clearance was obtained from Zayed University Research Ethics Committee (R201213) and from the Ministry of Health and Prevention's Research Ethics Committee (MOHAP/DXB-REC/MMM/No. 49/2020).

## Author Contributions

JT and FA led the concept and design and initial draft write up. SK and NE contributed to the discussion. All the authors contributed to writing the draft manuscript and read and agreed to the published version of the manuscript.

## Funding

This study was funded by the Cluster Grant R21001 awarded by Zayed University, United Arab Emirates. The funding body was not involved in the design of the study and collection, analysis, and interpretation of data or in writing the manuscript.

## Conflict of Interest

The authors declare that the research was conducted in the absence of any commercial or financial relationships that could be construed as a potential conflict of interest.

## Publisher's Note

All claims expressed in this article are solely those of the authors and do not necessarily represent those of their affiliated organizations, or those of the publisher, the editors and the reviewers. Any product that may be evaluated in this article, or claim that may be made by its manufacturer, is not guaranteed or endorsed by the publisher.
