# Supplementary material for: Outdoor Activity in the Daytime, but Not the Nighttime, Predicts Better Mental Health Status During the COVID-19 Curfew in the United Arab Emirates
Source: Front Public Health. 2022 Apr 4;10:829362. doi: 10.3389/fpubh.2022.829362 (PMC9013851; doi:10.3389/fpubh.2022.829362)
Supplement: Supplementary file 1 [file Table_1.pdf]

Supplementary Table 1. Timeline of Control and Prevention Measures during COVID-19 Lockdown in the UAE

Date	Emirate	Control and Prevention Measures
Mar 8	UAE	Closure of nurseries, schools and universities
Mar 14	Abu Dhabi	Enactment of remote working for non-key workers
Mar 17	UAE	Closure of religious worship venues (mosques/churches)
Mar 21	UAE	Closure of recreational spaces (i.e., beach, parks)
Mar 22	Dubai	Social distancing in retail outlets (1.5 m)
Mar 24	UAE	Closure of all malls and non-essential retail
Mar 25	UAE	Grounding of flights
Mar 26	UAE	3- day disinfection program and night curfew
Mar 28	Abu Dhabi	First testing center opens
Apr 5	Dubai	Enactment of movement permit requirements
Apr 7	Dubai	First drive-through testing center open
Apr 9	UAE	13 drive-through testing centers open
Apr 9	UAE	Places of worship to stay closed as Ramadan approaches
Apr 12	UAE	Home testing program for people of determination (special needs)
Apr 16	Dubai	Field hospital with capacity to treat 3,000 COVID-19 patients opened
Apr 28	Abu Dhabi	Mandatory COVD-19 test for mall employees before reopening
